# Something Is Changing in Viral Infant Bronchiolitis Approach

**DOI:** 10.3389/fped.2022.865977

**Published:** 2022-04-14

**Authors:** Paolo Bottau, Lucia Liotti, Eleonora Laderchi, Alessandra Palpacelli, Elisabetta Calamelli, Carlotta Colombo, Laura Serra, Salvatore Cazzato

**Affiliations:** ^1^Pediatric and Neonatology Unit, Imola Hospital, Imola, Italy; ^2^Pediatric Unit, Department of Mother and Child Health, Salesi Children's Hospital, Ancona, Italy; ^3^Postgraduate School of Pediatrics, University of Bologna, Bologna, Italy

**Keywords:** bronchiolitis, guideline, viral bronchiolitis phenotype, bronchodilator, valved holding chambers

## Abstract

Acute Viral Bronchiolitis is one of the leading causes of hospitalization in the first 12–24 months of life. International guidelines on the management of bronchiolitis broadly agree in recommending a minimal therapeutic approach, not recommending the use of bronchodilators. Guidelines, generally, consider bronchiolitis as a “unique disease” and this runs the risk of not administering therapy in some patients who could benefit from the use of bronchodilators, for instance, in those who will develop asthma later in their life and face first episode in the age of bronchiolitis. Today, there is growing evidence that bronchiolitis is not a single illness but can have different “endotypes” and “phenotypes,” based on age, personal or family history of atopy, etiology, and pathophysiological mechanism. There is evidence that some phenotypes of bronchiolitis are more strongly associated with asthma features and are linked to higher risk for asthma development. In these populations, possible use of bronchodilators might have a better impact. Age seems to be the main feature to suggest a good response to a bronchodilator-trial, because, among children > 6 months old with bronchiolitis, the presence of a subset of patients with virus-induced wheezing or the first episode of asthma is more likely. While waiting for new research to define the relationship between therapeutic options and different phenotypes, a bronchodilator-trial (using short-acting β2 agonists with metered-dose inhalers and valved holding chambers) seems appropriate in every child with bronchiolitis and age > 6 months.

## Introduction

Acute Viral Bronchiolitis is one of the leading causes of lower respiratory tract infection and hospitalization in the first 12–24 months of life (1, 2). Several guidelines have been published on the diagnosis and management of bronchiolitis; these guidelines have different reference ages in the diagnosis (<24 months in United States and <12 months in Europe), but basically agree in recommending a minimal therapeutic approach without the use of bronchodilators (3–6).

Accumulating evidence has revealed that bronchiolitis is not a single disease but can have different “endotypes” and “phenotypes” based on age of presentation, personal or family history of atopy, etiology, pathophysiological mechanism and clinical presentation (7–10). Therefore, today something seems changing in the viral infant bronchiolitis approach: in some phenotypes, drug treatment may be effective.

In this review, we try to address these topics, evaluating the different etiopathogenetic, clinical and therapeutic aspects of acute viral bronchiolitis.

## Pathogenetic Mechanisms

Bronchiolitis is more often described as inflammation and oedema of the bronchioles caused by respiratory viruses that invade the epithelial cells of the small airways. Although the exact mechanism is unknown, epithelial cells become necrotic and are sloughed off with an excessive amount of mucus. This condition leads to obstruction of the bronchioles and varying degrees of bronchospasm and air trapping (11).

Bronchiolitis is mostly associated with respiratory syncytial virus (RSV), that is detected in 50–80% of the hospitalized bronchiolitis cases (1, 12, 13). Other viruses can also be associated with bronchiolitis: rhinovirus (RV), human bocavirus, metapneumovirus, parainfluenza virus, influenza virus, adenovirus, coronavirus. Viral co-infections (usually with RSV and RV) are detected in 10–40% of severe cases (12). RSV is an RNA virus with two antigenically different subtypes (A with 11 genotypes and B with 23 genotypes) (14). RSV infection can be transmitted through direct inoculation of contaminated secretions in the nasal and conjunctival mucosa or by inhalation of large respiratory droplets (2, 15). Reinfections in young children are possible and are usually mild, although severe cases have been reported (16).

RSV binds to epithelial cells and replicates, causing epithelial necrosis and ciliary destruction. After being infected by RSV, the epithelial cells are sloughed to lower respiratory tract, where the virus infects the ciliated epithelial cells of the bronchioles mucosa and pneumocytes in the alveoli. Viral attachment to the target cell is mediated by F and G RSV surface glycoproteins. Epithelial cells necrosis results in the inflammatory response, by increasing the production of cytokines, including alarmins, chemokines, and growth factors. This inflammatory milieu attracts innate lymphoid cells, dendritic cells, and granulocytes to the site of infection (17, 18).

A strong induction of antiviral type I and type III interferons and interferon-induced genes is the primary response of the RSV infected mucosa, but RSV has developed the capacity to evade this innate interferon (INF) response through RSV-NS1/2 proteins, which are able to reduce both IFN-I and INF-III responses (19, 20). The disease state following RSV infection is associated with an increase in IL-17 production. This cytokine is more prominent in neonates, thus contributing to more severe clinical aspects (21, 22).

Intraluminal airway obstruction is caused by cellular infiltration of the peribronchiolar tissue, mucus overproduction, sloughing of infected epithelial cells and inefficient ciliary beating (15). Air trapping and varying degrees of lobar collapse can be caused by plugs composed of cellular debris and mucus in the bronchiole lumens (1). Viral clearance is permitted by innate and adaptive immune responses and the bronchiolar epithelium begins to regenerate within 3–4 days after the symptoms resolution. Currently, many studies indicate an association between RSV bronchiolitis and subsequent development of asthma (8).

Three RSV genotypes have been identified: NA1, ON1, and BA, respectively. The NA1 genotype infects young infants and is related to a more severe clinical course; the BA genotype is associated with eosinophilia and family history of asthma, while, in general, the ON1 genotype is associated with less severe symptoms (23). Interestingly, Harford et al. found that RSV infection resulted in dysregulation of β2-adrenergic receptor (β2AR) function, position and number. This dysregulation can explain the ineffectiveness of β2 agonists in treating obstruction in RSV infected patients (24).

The second most common virus causing bronchiolitis during infancy is RV. Its detection frequently occurs in children over 12 months of age. RV is an RNA Enterovirus belonging to the Picornaviridae family.

Lower respiratory tract infections are typically due to a RSV infection, particularly in very young children; on the contrary, RV infections are more common in slightly older children, in those with atopic predisposition in particular, and can cause severe wheezing (17). Lower respiratory tract infection is usually caused by RV type A, while severe wheezing is more commonly linked to RV type C infection (13, 25).

A partial defect in mucosal antiviral innate interferon responses may be related to a higher risk of severe RV infection and wheezing in young children with a family history of allergy and asthma. Cadherin-related family member 3 (CDHR3) has recently been identified as a unique receptor for RV-C (26), thus explaining the high pathogenic potential of RV-C. It is important to underline that an increased risk of childhood asthma has been associated with a polymorphism in the CDHR3 gene (27). RV infections seem to induce a milder epithelial inflammation than RSV, but in RV infections the expression of IFN type I decreases, with a predominant Th2 immune response (28).

Recently, a group of Italian researchers has demonstrated the presence of differing Th1/Th2 balance in patient hospitalized during the peak epidemic months or out of this period. The latter group was found to have a higher Th2 polarization in the immune response with a greater production of IL-4 and lower levels of IFNγ; therefore, they hypothesize the presence of two phenotypes of bronchiolitis: the first with RSV infection during the peak period and the second with a possible genetic predisposition to atopy and hospitalized during the non-peak season (29).

In conclusion, bronchiolitis is due to direct viral cytotoxic injury in conjunction with a robust host inflammatory response, but the relative contribution remains uncertain and is probably related to the type of virus involved and to the variability of the individual immune response. The role of the underlying genetic mechanisms is not yet clearly understood.

## Clinical Presentation

Diagnosis of acute bronchiolitis is clinical, supported by epidemiological and virological data. The term is generally applied to the first episode of wheezing in infants younger than 12–24 months of age (2, 5, 8). Peak incidence occurs between 3 and 6 months of age (1). After an incubation period of 4–6 days, infants show signs of upper respiratory tract infection (2): cough, runny nose, and fever are followed by lower respiratory distress characterized by nasal flaring, tachypnea, increased work of breathing with intercostal, subcostal or supraclavicular retractions, use of abdominal muscles and grunting in the next 1–3 days (1, 8). Multiple respiratory sounds can be heard: respiratory crackles and bilateral wheezing are typical (1). Acute viral bronchiolitis is a quite dynamic disease: clinical severity usually peaks around 3–5 days from the symptoms onset, and the minute-to-minute variation in clinical findings is characteristic, as mucus and debris are cleared from the airways by coughing or as the child becomes asleep or agitated; thus, several examinations are recommended (1). Clinical assessment is also possibly confounded by nasal congestion, the resolution of which, with nasal discharge, can help ascertain whether respiratory sounds come from lower respiratory tract (2). Fever can be present in almost 30% of infants and usually occurs early in the course of disease (30).

Most infants have a mild clinical form that resolves in 10–14 days and can be managed at home; cough usually resolves within 2–3 weeks (31). Infant aged <3 months, born pre-term or with cardiopulmonary (e.g., chronic lung disease or congenital heart disease), immunodeficiency or neuromuscular disorders are at greater risk of severe disease, with complications including apnoea or bacterial infection (32). In particular, preterm infants are at a higher risk of apnoea (with a reported rate from 1 to 24%) (2), especially if with a corrected age of <2 weeks, birth weight <2.3 kg, respiratory rate <30 or >70 at presentation, and SpO_2_ of 90% or less at presentation (33).

RSV infection is typically associated with an increased severity of presentation, while other viruses cause milder phenotypes (26, 34). In a retrospective cohort of previously healthy RSV-infected patients, respiratory failure was associated with lethargy, grunting, and a PaCO_2_ ≥ 65 mmHg at initial emergency department presentation (35, 36).

Hospitalization is usually recommended when children present with poor feeding, severe retractions, oxygen saturation of 92% or less, a respiratory rate higher then 60/min and in the presence of significant social risk factors (e.g., poor parental reliability or inadequate home environment) (35). Severity Scoring tools have been developed and validated to be used in clinical settings and can be useful for an objective measure. In general, these scores should be integrated with other measures and repeated to obtain clinical assessment to guide practical decisions (1, 7).

Finally, attempts have recently been made to characterize the clinical phenotype of bronchiolitis. Dumas et al. identified several clinical profiles from a multicenter study on children admitted to hospital with bronchiolitis: Profile A with high probability of RV etiology, history of wheezing and wheezing at presentation, eczema, and older age of the patient; profile B: wheezing at presentation, but no history of wheezing or eczema and high probability of RSV infection; profile C: the most severely ill group, with a longer hospital stay, and a high probability of RSV infection; profile D: the less severe illness, including non-wheezing children with a shorter length of hospitalization (26). A clinical respiratory assessment of the first bronchiolitis episode based on lung-X-rays, respiratory outcomes (hypoxemia, wheezing, and/or sub-costal retractions), nasal protein levels of antiviral and type 2 cytokines (IFNγ, IL-10, IL-4, IL-13, IL-1β, and TNFα) was conducted also by Arroyo et al. (37) in 2020 to define mild, hypoxemia or wheezing phenotypes.

These studies provide informations about outcomes, disease patterns and underlying airway immunobiology and, above all, they may be the evidence of the need of a tailored clinical and therapeutic approach (8, 27).

## Therapeutic Options

Among international guidelines, there is broad agreement on the role of support therapy; it is well-established that bronchiolitis management should be focused on guarantying proper hydration and oxygenation of the child. Intravenous or nasogastric fluid administration is recommended to ensure hydration whenever oral route is non-viable. Moreover, the enteral route should be preferred to the intravenous route (1). Oxygen therapy is recommended in children with peripheral oxygen saturation below 92% (1, 38) and should be performed through standard oxygen therapy (SOT) or *via* high flow nasal cannula (HFNC), providing heated and humidified air (39, 40).

Drug administration is ground for controversy, as corticosteroids, nebulized hypertonic saline, and nebulized epinephrine are mostly not recommended, while β2 agonist bronchodilators are contemplated in some guidelines and object of a long-lasting debate (1, 41–43). Although several guidelines advise against bronchodilators administration (4–6), in some countries, such as in Italy (3), the β2 agonist trial is considered as a possibility in selected cases as well. The recommendation of some guidelines on minimal handling of bronchiolitis (3–6) may result in several concerns of pediatric care providers and in the lack of adherence to the guidelines themselves (7, 44).

The indication on the utility of bronchodilators is mainly based on Gadomski and Scribani Chochrane systematic review, which states that albuterol administration does not result in a significant reduction in hospitalization or disease duration in non-hospitalized children. However, these same authors point out that some children may benefit from the administration of bronchodilators (45). As Wall suggests (42), it may be possible that some children, who will develop asthma later in their life, may experience their first episode in the age range of bronchiolitis and, thus, be indistinguishable from children with “pure” bronchiolitis (4, 42). This could be the reason why older patients (>6–12 months) with history of personal or familiar atopy, moderate to severe respiratory distress and wheeze as predominant auscultatory feature (i.e., no crackles) may improve with an asthma type therapy (42). Common practice as well suggests that clinical features of bronchiolitis in infants under 3 months of age are quite different from infants 8–12 months old. These data have been confirmed by two Italian studies, that noted how bronchiolitis in infants under 6 months of age is different from that in infants older than 6 months (46, 47).

Recent evidence noted that several pathogenetic clusters based on age, viral agents, immune phenotype, presence of wheeze and crackles, disease severity and risk of recurrent wheezing and asthma may underlie bronchiolitis (7–10). One group may be formed by very young infants (<6 months of age) with RSV infection and increased risk of recurrent wheezing; instead, another group may comprise older children (>6 months of age) with RV infection, atopic predisposition, high risk of developing asthma (8) and, probably, a better response to bronchodilators. Although much more evidence is required, proposals were put forward for a more personalized treatment, based on the hypothesis of different entities of bronchiolitis (8, 48). An interesting recent study by Rodríguez-Martínez et al. (49) found out that therapeutic options based on a phenotypic-guided strategy are also more cost-effective than a guideline-guided strategy.

Considering all these data, there is evidence that some phenotypes of bronchiolitis are more strongly associated with asthma features and are linked to higher risk for asthma development. In these populations, use of bronchodilators might have a better impact. More specifically, we propose that an inhaled bronchodilator trial could be considered particularly in children older than 6 months of age, but two specific conditions must be advised. The first is that in these infants, that we can consider as “viral wheezers,” bronchodilators should be administrated by metered-dose inhalers and valved holding chambers, as recommended by current guidelines, since this method is more effective and with less side effects than nebulized therapy (50, 51). The second is the indication to observe a clinical improvement with clinical scores after the bronchodilator trial (52). The dose and the time when to re-evaluate the clinical picture are still debated, perhaps the treatment scheme of worsening asthma in children younger than 5 years proposed by the GINA 2021 guidelines (short-acting β2 agonist: 2–4 puff every 20′ for three times in the first hour and review) can be applied (51).

Similarly to bronchodilators, the use of corticosteroid is not recommended in the management of infant bronchiolitis (1), but is part of the treatment of viral wheezing or asthma, so we agree that a course of corticosteroid could be indicated in moderate to severe clinical pictures, when the bronchodilators trial gave a positive result (42, 53–56).

These several data and considerations stress the necessity of subtype-specific studies, in order to evaluate the clinical response to different treatments and meet the need for a more personalized precision therapy.

## Discussion and Conclusion

Bronchiolitis is one of the major health problems in infants around the world, but unfortunately, to date, no therapy has been validated for the management of this disease. Many guidelines have been published by various scientific societies, which agree on minimal handling (i.e., hydration and the administration of oxygen as the only treatment options) (3–6). Unfortunately, this approach risks excluding some patients who might benefit from the bronchodilator treatment (48). However, as pointed out by the American Academy of Pediatrics, guidelines are intended to assist clinicians in decision making and are not intended to “*replace clinical judgment or establish a protocol for the care of all children with bronchiolitis*” (5). In light of the data emerging from the scientific literature, acute bronchiolitis should no longer be considered as a single disease but rather as characterized by different phenotypes and endotypes (7–10, 26). In particular, it seems that the phenotype characterized by older age, type 2 immune response, personal or family (first-degree) history of atopy, RSV genotypes (ON1 and BA) or RV etiology and infection during non-RSV-predominant or non-peak viral months may have a good response to a trial with bronchodilators (48).

Evaluating all the evidence, age seems to be the main feature that may suggest a good response to the bronchodilator-trial. For instance, in the research by Dumas et al., the “profile A” (patient with personal or family history of wheezing and eczema, wheezing at clinical presentation, and RV infection) is often >6 months, probably have initial sign of asthma and may respond to a short-acting β2 agonist-trial (26, 57). Probably, the “true” bronchiolitis (in which therapy is useless) affects the child <6 months. This hypothesis may be supported by epidemiological data (1, 4), showing that bronchiolitis reaches its peak of incidence between 3 and 6 months, and by other studies suggesting that bronchiolitis in infants under 3–6 months of age is quite different from that in infants >6 months old (46, 47).

In a recent multicenter study with the objective of identifying factors associated with the use of albuterol among infants hospitalized for bronchiolitis, Condella et al. found that there is a subgroup of patients to whom clinicians preferentially administer albuterol. Characteristics of these infants mainly include presentation with wheeze and older age (>6 months), and these children may be similar to those affected by viral wheezing or their first episode of asthma (58).

This knowledge suggests that among infants >6 months with bronchiolitis, there could be a subset of patients who may have a virus-induced wheezing or their first episode of asthma and may respond to the bronchodilator-trial (4, 42).

To conclude, there is evidence that some phenotypes of bronchiolitis are more strongly associated with asthma features and are also linked to higher risk for asthma development. In these populations, use of bronchodilators might have a better impact.

Looking forward to future RCTs targeted to assess the utility of short-acting β2 agonists in different bronchiolitis phenotypes, a bronchodilator-trial (using short-acting β2 agonists with metered-dose inhalers and valved holding chambers) could be performed in infants with bronchiolitis and age > 6 months.

## Author Contributions

PB and LL designed the review. PB, LL, EL, AP, and CC performed literature search and draft the manuscript. PB, LL, and EC review and editing. LS and SC supervision the manuscript. All authors contributed to the article and approved the submitted version.

## Conflict of Interest

The authors declare that the research was conducted in the absence of any commercial or financial relationships that could be construed as a potential conflict of interest.

## Publisher's Note

All claims expressed in this article are solely those of the authors and do not necessarily represent those of their affiliated organizations, or those of the publisher, the editors and the reviewers. Any product that may be evaluated in this article, or claim that may be made by its manufacturer, is not guaranteed or endorsed by the publisher.
